# WNK1 kinase is essential for insulin‐stimulated GLUT4 trafficking in skeletal muscle

**DOI:** 10.1002/2211-5463.12528

**Published:** 2018-10-05

**Authors:** Ji‐Hee Kim, Hanul Kim, Kyu‐Hee Hwang, Jae Seung Chang, Kyu‐Sang Park, Seung‐Kuy Cha, In Deok Kong

**Affiliations:** ^1^ Department of Physiology Yonsei University Wonju College of Medicine Korea; ^2^ Department of Global Medical Science Yonsei University Wonju College of Medicine Korea; ^3^ Mitohormesis Research Center Yonsei University Wonju College of Medicine Korea; ^4^ Institute of Lifestyle Medicine Yonsei University Wonju College of Medicine Korea; ^5^ Institute of Mitochondrial Medicine Yonsei University Wonju College of Medicine Korea

**Keywords:** diabetes mellitus, GLUT4, insulin, skeletal muscle, trafficking, WNK1

## Abstract

With‐no‐lysine 1 (WNK1) kinase is a substrate of the insulin receptor/Akt pathway. Impaired insulin signaling in skeletal muscle disturbs glucose transporter 4 (GLUT4) translocation associated with the onset of type 2 diabetes (T2D). WNK1 is highly expressed in skeletal muscle. However, it is currently unknown how insulin signaling targeting WNK1 regulates GLUT4 trafficking in skeletal muscle, and whether this regulation is perturbed in T2D. Hereby, we show that insulin phosphorylates WNK1 at its activating site via a phosphatidylinositol 3‐kinase‐dependent mechanism. WNK1 promotes the cell surface abundance of GLUT4 via regulating TBC1D4. Of note, we observed insulin resistance and decreased WNK1 phosphorylation in T2D *db/db* mice as compared to the control mice. These results provide a new perspective on WNK1 function in the pathogenesis of hyperglycemia in T2D.

**Abbreviations**
AS160: akt substrate of 160 kDa
GCM: gastrocnemius
GLUT4: glucose transporter 4
IGF‐1R: insulin‐like growth factor‐1 receptor
InsR: insulin receptor
PI3K: phosphatidylinositol 3‐kinase
SNARE: soluble NSF attachment protein receptor
T2D: type 2 diabetes
TBC1D4: TBC1 domain family member 4
WNK1: with‐no‐lysine 1

With‐no‐lysine [K] (WNK) kinases are a family of serine/threonine protein kinases with an atypical placement of the catalytic lysine 1, 2. Mammalian WNK kinases consist of four members (WNK1‐4) with tissue‐specific expression 3. The mutations of WNK1 and WNK4 were shown to cause human genetic disease, the pseudohypoaldosteronism type 2 (also known as familial hyperkalemic hypertension or Gordon's syndrome) whose clinical phenotypes are characterized by hypertension and hyperkalemia 4. WNK1 and WNK4 are the master regulators of ion homeostasis in the kidney and brain 3, 5. WNK1 is widely expressed and regulates various ion channels and transporters 3, 5. While the mechanism and mediators of WNK1‐mediated epithelial ion transports are relatively well established, little is known about its extrarenal function beyond electrolyte homeostasis.

With‐no‐lysine 1 is ubiquitously expressed including skeletal muscles 3, 6, which are the major tissue of glucose metabolism. Insulin is a primary physiological stimulus for glucose uptake in skeletal muscles 7. Insulin signaling activates glucose transporter 4 (GLUT4) for its translocation to the plasma membrane, thus stimulating glucose uptake through phosphatidylinositol 3‐kinase (PI3K)/Akt signaling cascades 7, 8, 9. TBC1 domain family member 4 (TBC1D4) (also known as AS160), the Akt substrate (AS), promotes soluble NSF attachment protein receptor (SNARE)‐associated GLUT4 trafficking upon insulin stimulation 8, 9. WNK1 is also a substrate of Akt, a downstream target of insulin receptor (InsR)–PI3K pathway which stimulates endocytosis of ion channels and transporters to regulate ion homeostasis 3, 10, 11, 12. Impaired insulin‐responsive GLUT4 translocation in skeletal muscle is associated with the onset of type 2 diabetes (T2D) 7, 8. In fact, WNK1 is one of the highly expressed isoforms of WNK kinases in skeletal muscle 3, 6 and is known to regulate GLUT1 trafficking via regulating TBC1D4 in HEK293 cells 13. However, it is currently unknown whether insulin signaling targeting WNK1 regulates GLUT4 translocation in skeletal muscle, and whether InsR–WNK1 signaling is impaired in T2D.

In this study, we show that a highly expressed WNK1 regulates insulin‐stimulated GLUT4 trafficking in mouse skeletal muscle cells. Moreover, InsR/Akt/WNK1 signaling cascades are impaired in T2D *db/db* mice. We demonstrate a new perspective on the biological function of WNK1 in skeletal muscles beyond the regulation of ion transport and on the clues for the pathogenesis of hyperglycemia in T2D.

## Materials and methods

### Reagents

Unless otherwise noted, all chemicals and reagents including insulin (Cat no. I2643) were purchased from Sigma‐Aldrich (St Louis, MO, USA). 2‐(4‐morpholinyl)‐8‐phenylchromone (LY294002) (Cat no. 19–142) was purchased from Calbiochem (San Diego, CA, USA).

### Animal models

C57BLKS/6J *db/m* and *db/db* male mice (*n* = 6 each) aged 20 weeks were obtained from Japan Shizuoka Laboratory Center (Shizuoka, Japan). *db/m* mice were designated as a control group. All mice were housed in the individual ventilated cage (IVC) racks at a constant temperature (22 ± 3 °C) and relative humidity (50 ± 10%) using fluorescent lamps (lights were on 6:00–18:00) for 12 h and fed with solid feed 5L79^®^ (LabDiet, St. Louis, MO, USA). Throughout the experiment, they were examined for blood glucose, body weight, and food intake per week during maintaining. All approvals for animal study were made by the Yonsei University Wonju College of Medicine Institutional Animal Care and Use Committee (YWC‐130826‐2).

### Cell culture, transfection, and knockdown by small interfering RNA

C2C12  myoblasts and L6 muscle cells expressing c‐myc epitope‐tagged GLUT4 (L6‐GLUT4) were provided from the American Type Culture Collection (ATCC, Manassas, VA, USA) and H. S. Kim (Korea University, Korea), respectively. Both C2C12 and L6‐GLUT4 were grown in Dulbecco's modified Eagle medium (DMEM, Cat no. SH30243, Hyclone, Logan, UT, USA) containing 10% (v/v) (FBS, Cat no. SH30919.03, Hyclone), 100 units·mL^−1^ penicillin and 100 μg·mL^−1^ streptomycin (Cat no. SV30010, Hyclone) with a humidified atmosphere consisting of 95% air and 5% CO_2_ at 37 °C. At 80–90% confluence, the differentiation of the cells into multinuclear myotubes was facilitated by switching the culture media to DMEM containing 5% and 2%) horse serum (Cat no. 26050‐088, Gibco, Grand Island, NY, USA) for C2C12 and L6‐GLUT4, respectively, 100 units·mL^−1^ penicillin and 100 μg·mL^−1^ streptomycin every 24 h for 5–7 days. HEK293FT (human embryonic kidney 293 stably expressing the SV40 large T antigen) cells were cultured as previously described 14. DNA constructs of myc‐tagged WNK1 (1‐491), WNK1 (T58A), and WNK1 (K233M) were previously described 15, 16. All DNA plasmids were transfected by using X‐tremeGENE HP DNA transfection reagent^Ⓡ^ (Cat no. 06 366 236 001, Roche, Mannheim, Germany) according to the manufacturers’ instructions. Experiments were conducted 48 h after transfection. On the 3rd day after switching the media, C2C12 was transfected with nontargeting control oligonucleotide (Cat no. sc37007), and mouse siRNA for WNK1 (Cat no. sc39257) and TBC1D4 (Cat no. sc61655) were provided from Santa Cruz Biotechnology (Santa Cruz Biotechnology, Santa Cruz, CA, USA). Transfection of siRNA oligonucleotide was performed using DharmaFECT siRNA transfection reagent (Cat no. T‐2001‐03, Thermo Scientific, Lafayette, CO, USA) as per manufacturer's instructions. Knockdown of genes and signaling effects were assessed 96 h after transfection by determining the protein levels using immunoblotting.

### Reverse transcription PCR (RT‐PCR) and quantitative real‐time PCR (qPCR)

Purified total RNA were acquired from the trypsinized pellets of C2C12 and mouse gastrocnemius (GCM) tissue from *db/m* and *db/db* through Hybrid‐R™ total RNA purification kit (Cat no. 305–101, GeneAll, Seoul, Korea). Complementary DNA (cDNA) was made from 1 μg of total RNA via a ReverTraAce® qPCR RT Master Mix with gDNA Remover (Cat no. FSQ‐301, Toyobo, Osaka, Japan). The mRNA abundance was analyzed by quantitative real‐time PCR with SYBR‐green (204143, Qiagen, Hilden, Germany) using sequence‐specific primers: WNK1, forward 5′‐ACCTAGTGTACCTGCAGTGGTG‐3′, reverse 5′‐TTGCTGAGACACC TGGGAAG‐3′; WNK2, forward 5′‐ATGACTTCTGGGAGTCCAGT‐3′, reverse 5′‐CCGCTTCAGGTACGTCTTCAGT‐3′; WNK3, forward 5′‐GCCAAAATATCCACCTCCTGTC‐3′, reverse 5′‐ GTTTACATGAGATGTCCCCAGATG‐3′; WNK4, forward 5′‐TCATGGCTCCTGAGATGTAC‐3′, reverse 5′‐CATGCACATGCCAAAGGCGTAC‐3′; myogenic differentiation 1 (Myod1), forward 5′‐ ATCGCATTGGGGTTTGAGCC‐3′, reverse 5′‐GGCATGATGGATTACAGCGG‐3′; Myogenin, forward 5′‐TCCACGATGGACGTAAGGGA‐3′, reverse 5′‐CATGGTGCCCAGTG AATGCA‐3′; β‐actin, forward 5′‐AAGAGCTATGAGCTGCCTGA‐3′, reverse 5′‐CACAG GATTCCATACCCAAG‐3′; 18s, forward 5′‐AACCCGTTGAACCCCATT‐3′, reverse 5′‐CCATCCAATCGGTAGTAGCG‐3′. For the analysis of each gene expression, experiments were conducted in triplicate with real‐time PCR system (7900HT, Applied Biosystems, Foster City, CA, USA). Data were analyzed by 2^**−ΔΔCT**^ method with 18s (18s ribosomal RNA subunit) as a reference gene. The primers used for qPCR were the same as those designed for RT‐PCR.

### Western blot and cell surface biotinylation assay

For biotinylation of cell surface GLUT4, cells were washed with ice‐cold PBS three times and incubated with 0.75 mL chilled PBS containing 1.5 mg·mL^−1^ EZ‐link NHS‐SS‐biotin (Thermo Scientific) for 1 h at 4 °C. After quenching with glycine‐containing PBS (100 mm), cells were lysed in a RIPA buffer (150 mm NaCl, 50 mm Tris/HCl, 5 mm EDTA, 1% Triton X‐100, 0.5% deoxycholate, and 0.1% SDS) containing protease inhibitor mixture. Biotinylated proteins were precipitated by streptavidin‐agarose beads for overnight at 4 °C. The beads were subsequently washed four times with PBS containing 1% Triton X‐100. Biotin‐labeled proteins were eluted in the sample buffer, separated by SDS/PAGE, and transferred to poly(vinylidene difluoride) (PVDF) membranes for western blotting. Blots were developed using enhanced chemiluminescence. Biotinylation experiment was performed 3–4 times with similar results. Primary antibodies used western blots for p‐IGF‐1Rβ^Thr1131^/InsRβ^Tyr1146^ (1 : 1000 dilution, Cat no. 3021), InsR (1 : 1000 dilution, Cat no. 3025), p‐Akt^Ser473^ (1 : 2000 dilution, Cat no. 9271), p‐Akt^Thr308^ (1 : 2000 dilution, Cat no. 2965), Akt (1 : 2000 dilution, Cat no. 9272), p‐TBC1D4^Ser588^ (1 : 1000 dilution, Cat no. 8730), TBC1D4 (1 : 1000 dilution, Cat no. 2670), and GLUT4 (1 : 2000 dilution, Cat no. 2213) were provided from Cell Signaling Technology (Beverly, MA, USA). Phospho‐WNK1 antibody detecting at threonine58 (T60 for human; 1 : 1000 dilution, Cat no. STJ90776) was purchased from St John's Laboratory (London, UK). β‐actin (1 : 10000 dilution, Cat no. ab6276), WNK1(1 : 1000 dilution, Cat no. ab128858), and myc‐HRP (1 : 5000 dilution, Cat no. ab1326) were from Abcam (Cambridge, MA, USA). GAPDH (1 : 3000 dilution, Cat no. sc25778) were purchased from Santa Cruz Biotechnology. Relative protein levels were normalized by internal controls, GAPDH or β‐actin in western blot analysis. Densitometry for western blot bands was analyzed using ImageJ software (version 1.8, National Institutes of Health, Bethesda, MD, USA).

### Statistical analysis

Data analysis was performed with the Prism software (version 6, graphpad Software, San Diego, CA, USA). Statistical comparisons between two groups were made using a two‐tailed unpaired Student's *t*‐test. Multiple comparisons were determined using one‐way ANOVA followed by Tukey's multiple comparison tests. *P‐*values < 0.05 and 0.01 were considered significant for single and multiple comparisons, respectively. All data were presented as the mean ± SEM.

## Results

### WNK1 is a downstream substrate of insulin signaling pathway in C2C12 myotubes

**Figure 1:**
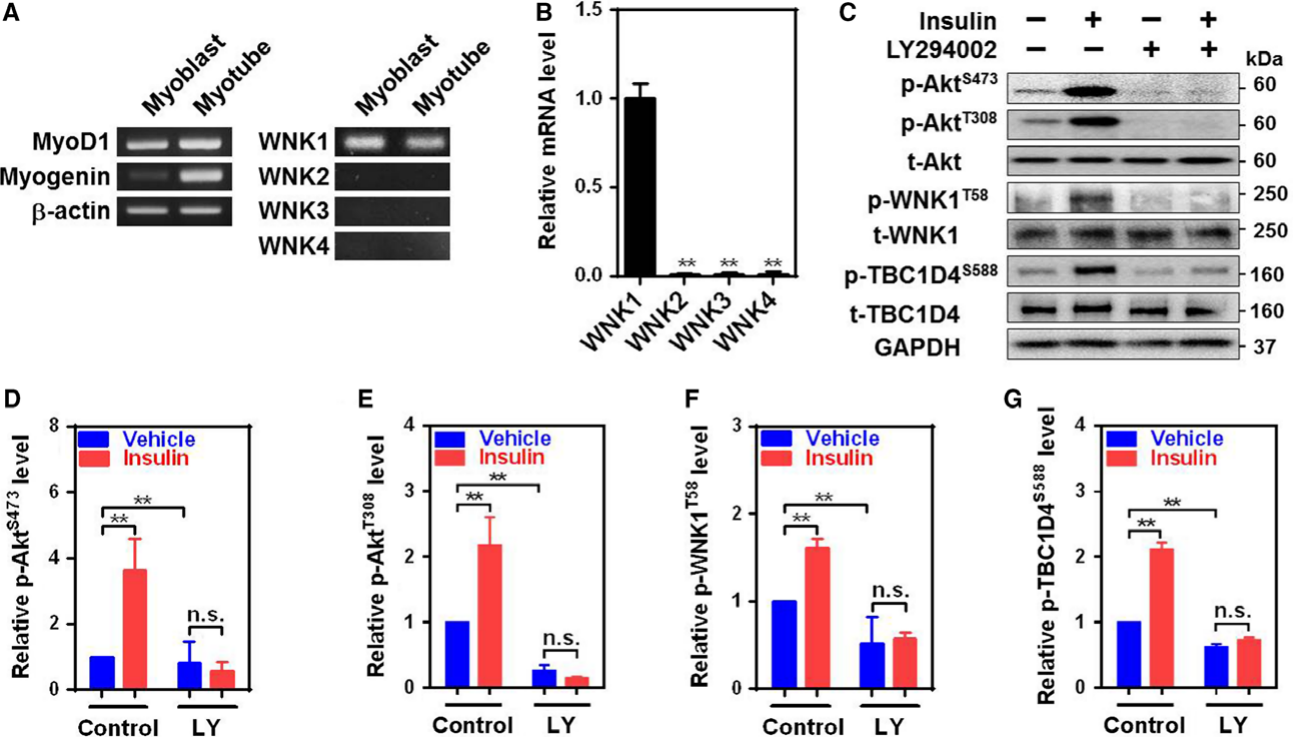
WNK1 is a mediator of insulin signaling in C2C12 mouse skeletal muscle cells. (A) RT‐PCR analysis showing the validation of *in vitro* C2C12 myotubes differentiation and expression of WNK kinases. Differentiation of C2C12 myoblasts into myotubes was confirmed by myogenin and myogenic differentiation 1 (MyoD1) expression (left). Expression of WNK1‐4 in myotubes and myoblasts (right). (B) Quantitative real‐time PCR for WNK1‐4 in C2C12 myotubes. **P <* *0.01 vs WNK1. (C) Immunoblotting showing the effect of PI3K on InsR signaling pathways with the following markers: phospho‐Akt at serine473 (p‐Akt^S473^) and at threonine308 (p‐Akt^T308^); total Akt (t‐Akt); phospho‐WNK1 (p‐WNK1^T58^); total WNK1 (t‐WNK1); phospho‐TBC1D4 at serine588 (p‐TBC1D4^S588^); and total TBC1D4 (t‐TBC1D4). Insulin (100 nm, for 15 min) or LY294002 (50 μm, for 30 min), a PI3K inhibitor, were treated into C2C12 cells. GAPDH was served as a loading control. (D–G) Quantification of protein levels of p‐Akt^S473^ (D), p‐Akt^T308^ (E), p‐WNK1^T58^ (F), and p‐TBC1D4^S588^ (G) was shown from panel C. All protein levels were normalized by GAPDH. LY, LY20 ‐‐> 94002. All values are expressed as the mean ± SEM and **P <* *0.01. Experiments were repeated 3–4 times with similar results and one‐way ANOVA (B and D–G).

Skeletal muscle is one of the major metabolic tissues for uptaking glucose which is stimulated by InsR signaling cascades including PI3K, Akt, and TBC1D4 7, 8, 9. WNK1 is widely expressed in various tissues including skeletal muscle 3, 6. While skeletal muscle expresses a high level of WNK1 as compared with other WNK kinases, the upstream regulators and downstream targets of WNK1 signaling have been relatively unexplored. We used C2C12 skeletal muscle cells as an *in vitro* model to study WNK1 expression and signaling cascades (Fig. 1A). Among WNK kinases, WNK1 is highly expressed in C2C12 myotubes (Fig. 1A,B). WNK1 at threonine58 (T58; T60 for human) is phosphorylated by Akt kinase 11, 12, which is a downstream target of InsR–PI3K activation. We explored WNK1 as a downstream signaling molecule of insulin activation in skeletal muscle cells. Consistent with previous reports 10, 11, 12, insulin phosphorylated Akt and its downstream substrates, WNK1 and TBC1D4, via PI3K activation in C2C12 myotubes (Fig. 1C–G). InsR–Akt pathway regulates TBC1D4 to stimulate the translocation of GLUT4 to plasma membrane 9, 17, 18, 19. Together, these data support the notion that WNK1 may contribute to GLUT4 translocation by the stimulation of InsR signaling cascades.

### Insulin stimulates GLUT4 translocation via PI3K‐ and WNK1‐dependent mechanism

**Figure 2:**
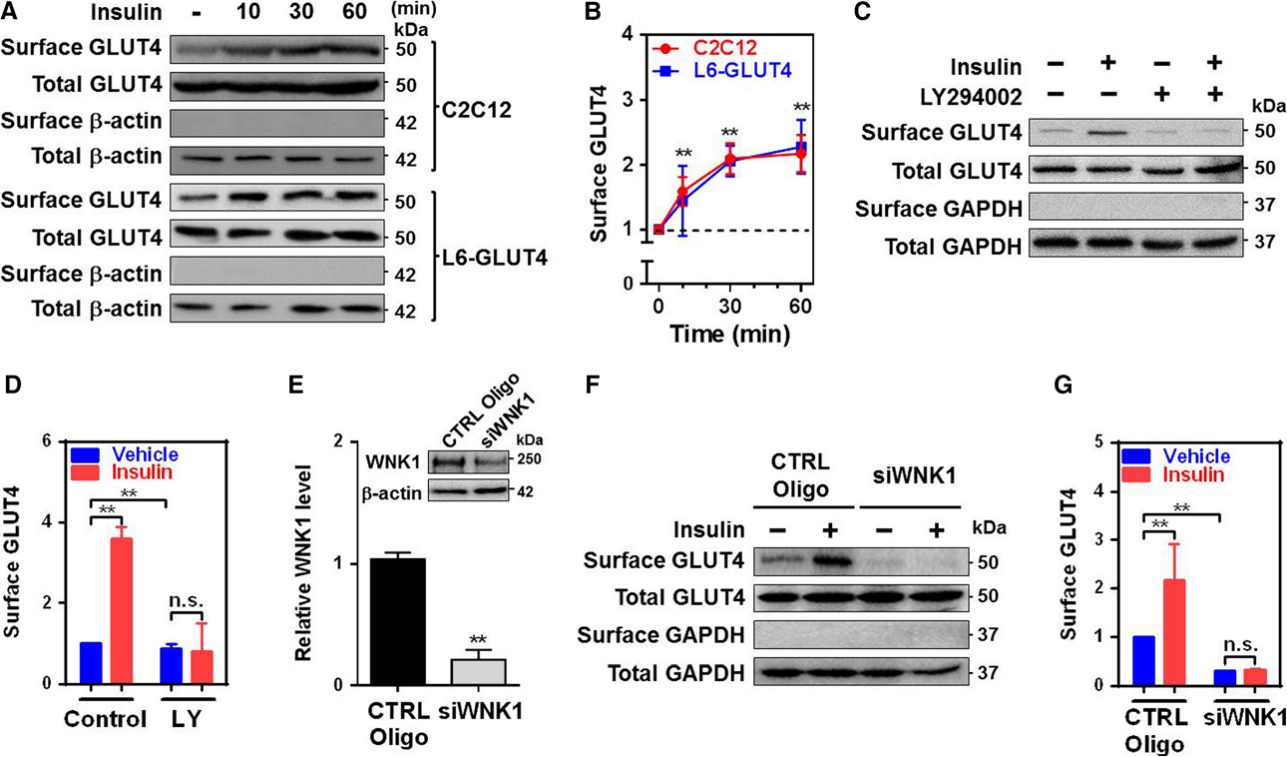
Insulin promotes the cell surface abundance of GLUT4 via PI3K‐ and WNK1‐dependent mechanism. (A) Time‐dependent effect of insulin on GLUT4 translocation in C2C12 and GLUT4 stably expressing L6 (L6‐GLUT4) myotubes. The cell surface abundance of GLUT4 was evaluated by biotinylation assay. (B) Densitometry analysis of the results in panel A. (C) Cell surface biotinylation assay showing the effect of PI3K inhibitor, LY294002 (50 μm, for 30 min), on the cell surface expression of GLUT4 by insulin stimulation (100 nm, 1 h) in C2C12 myotubes. (D) Densitometry analysis of the results in panel C. n.s., not significant. (E) Validation of WNK1 siRNA knockdown. (F) Effect of WNK1 siRNA on insulin‐promoted GLUT4 translocation in C2C12 myotubes. Specific biotinylation of membrane GLUT4 was supported by the lack of the GAPDH detection in the membrane fraction in all experiments. Control oligonucleotides (CTRL Oligo) are a nontargeting control siRNA. GAPDH was used as loading controls. (G) Densitometry analysis of the results in panel F. All values are the mean ± SEM and **P <* *0.01. All protein levels were normalized by internal controls, β‐actin (B,E) or GAPDH (D,G). Experiments were repeated 3 times with similar results Student's *t*‐test (E) and one‐way ANOVA (B,D, and G).

WNK1 regulates both endocytosis and exocytosis of various transmembrane transporter proteins 3, 5. WNK1 promotes SNARE‐associated vesicle delivery and fusion to plasma membrane 8, 13, 20, 21. Insulin activates PI3K/Akt kinase to promote the translocation of SNARE‐associated GLUT4 vesicle 8, 9. Thus, we examined whether WNK1 stimulates the cell surface expression of GLUT4 in skeletal muscle cells via PI3K‐dependent mechanism. Insulin treatment promoted the cell surface abundance of GLUT4 in C2C12 and L6‐GLUT4 myotubes in a time‐dependent manner (Fig. 2A,B). The cell surface abundance of GLUT4 stimulated by insulin was blunted by PI3K inhibitor treatment (Fig. 2C,D). Moreover, WNK1 siRNA knockdown decreased basal and insulin‐stimulated cell surface abundance of GLUT4 (Fig. 2E–G), indicating that WNK1 regulates constitutive translocation of GLUT4 at the basal state and insulin‐stimulated cell surface abundance of GLUT4. Together, these data suggest that insulin promotes the cell surface expression of GLUT4 via PI3K‐ and WNK1‐dependent mechanism.

### WNK1 kinase activity contributes to insulin‐mediated TBC1D4 phosphorylation

**Figure 3:**
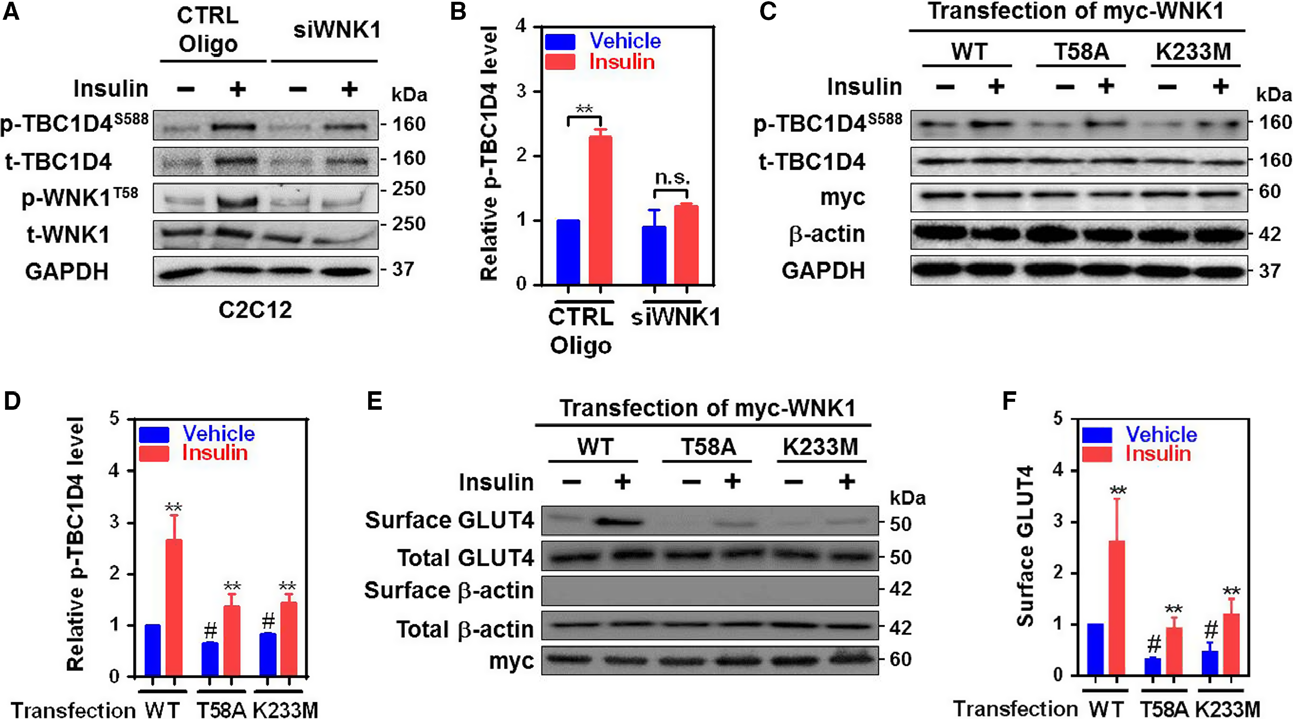
WNK1 kinase activity is critical for insulin‐induced TBC1D4 activation. (A) Effect of WNK1 siRNA on TBC1D4 phosphorylation in C2C12 myotubes. Insulin (100 nm, 15 min) treatment was followed after serum deprivation. (B) Summary of the results in panel A. n.s., not significant. (C) Effect of WNK1 mutants of Akt phosphorylation defect (T58A) and kinase‐dead (K233M) on insulin (100 nm, 15 min)‐induced TBC1D4 phosphorylation. WNK1 constructs were transfected in HEK293FT cells. WT, wild‐type. (D) Summary of the results in panel C. (E) Cell surface biotinylation assay showing the effects of WNK1 constructs (WT, T58A or K233M) on insulin‐stimulated cell surface expression of GLUT4 in HEK293FT. Insulin (100 nm, 1 h) treatment was followed after serum deprivation. (F) Summary of the results in panel E. The relative surface GLUT4 was normalized with total GLUT4 and β‐actin. GAPDH (A,C) or β‐actin (C,E) was used as loading controls. All values are expressed as the mean ± SEM. **P <* *0.01 vs Vehicle; ^#^ *P *<* *0.05 vs WT treated with vehicle. All protein levels were normalized by internal controls, GAPDH (B,D) or β‐actin (F). Experiments were repeated 3 times independently with similar results and one‐way ANOVA (B,D, and F).

Akt phosphorylates its substrate TBC1D4 to stimulate the translocation of GLUT4 to the plasma membrane in skeletal muscle and adipose tissues 8, 17. WNK1 is also an AS 11, 12 which phosphorylates TBC1D4 to regulate GLUT1 trafficking in HEK293 cells 13. Therefore, we examined whether WNK1 regulates insulin‐induced TBC1D4 phosphorylation. As shown in Fig. 3A,B, TBC1D4 phosphorylation by insulin was blunted by WNK1 siRNA knockdown in C2C12 myotubes. Insulin activates Akt to phosphorylate WNK1 at threonine58 (T58) by regulating membrane retention of channel proteins 10, 22. To examine the role of kinase activity and Akt phosphorylation of WNK1, we used HEK293FT cell heterologously expressing Akt phosphorylation defect (T58A) or kinase‐dead (K233M) WNK1 mutants because of low transfection efficiency of C2C12 myotube. Coexpression with WNK1 mutants (T58A or K233M) inhibited basal and the insulin‐stimulated TBC1D4 phosphorylation (Fig. 3C,D), indicating that WNK1‐induced TBC1D4 regulation occurs in the basal state and in response to insulin stimulation. This supports that WNK1 is an important regulator of TBC1D4. Moreover, insulin‐stimulated GLUT4 translocation was also reduced by overexpression of WNK1 mutants, T58A or K233M (Fig. 3E,F). In this regard, these data indicate that WNK1 is a key regulator of TBC1D4 to stimulate the surface expression of GLUT4 in skeletal muscle.

### WNK1 signaling is impaired in diabetic skeletal muscle

**Figure 4:**
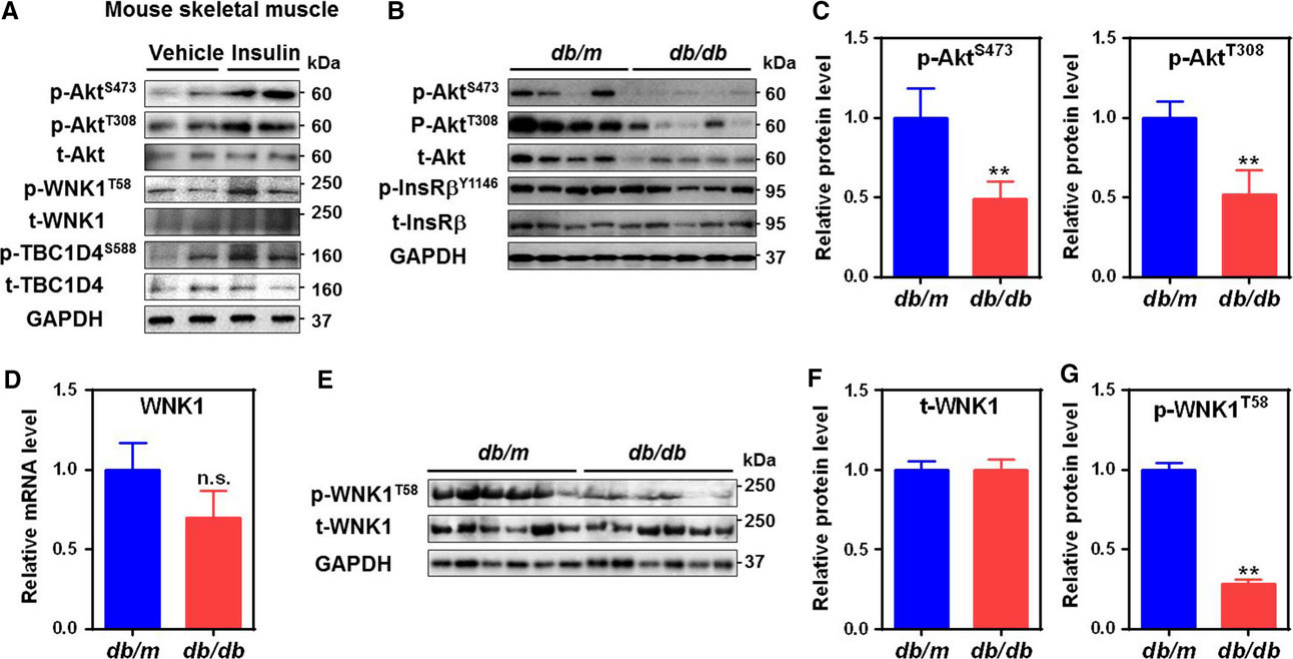
Insulin‐WNK1 signaling pathway is blunted in diabetic skeletal muscle. (A) Immunoblotting showing the effect of insulin administration (i.p. injection of insulin (5 U·Kg^−1^)) on InsR downstream targets related to GLUT4 translocation in mouse skeletal muscle (GCM muscle; GCM). (B) InsR signaling in *db/m* and *db/db* mice (*n* = 4–5). Immunoblotting was performed by using GCM. (C) Quantification of Akt phosphorylation; p‐Akt^S473^ (left) and p‐Akt^T308^ (right) by densitometry in panel B. (D) Quantitative real‐time PCR for WNK1 in GCM of *db/m* and *db/db* mice (*n* = 6 each). n.s., not significant. (E) Representative immunoblotting showing both phosphorylated and total amounts of WNK1 protein from *db/m* and *db/db*. (F–G) Quantification of total (F; t‐WNK1) and phospho‐WNK1 (G; p‐WNK1^T58^) from panel E. Data points are expressed as the mean ± SEM and analyzed by Student's *t*‐test (C,D,F, and G). Experiments were repeated 3 times with similar results **P <* *0.01. GAPDH was used as a loading control (A,B, and E) .

The above results demonstrate that WNK1 promotes GLUT4 translocation by the activation of InsR signaling cascades in C2C12 myotubes *in vitro*. Next, we examined WNK1 as a downstream target of insulin signaling cascade in skeletal muscle *in vivo*. Insulin infusion increased phosphorylation of WNK1 in skeletal muscle as well as that of Akt and TBC1D4 (Fig. 4A). Insulin resistance and impairment of glucose uptake in skeletal muscle are primary clinical manifestations of T2D 7, 8. Subsequently, we examined insulin resistance and WNK1 signaling in *db/db* mice skeletal muscle. These mice are known to progress T2D and have very high levels of plasma glucose level and HOMA‐IR 7. Consistent with previous studies 7, phosphorylation of InsR and Akt has been markedly blunted supporting that insulin signaling is impaired in skeletal muscle of *db/db* mice (Fig. 4B,C). We then examined WNK1 signaling in T2D skeletal muscle. While the expression levels of mRNA and total WNK1 protein were not altered (Fig. 4D–F), WNK1 phosphorylation was significantly blunted (Fig. 4E,G) suggesting that WNK1 activity, but not its expression, is reduced in T2D skeletal muscle. Taken together, these results support that InsR–Akt–WNK1 cascade is impaired in diabetic skeletal muscle resulting in the onset of T2D in skeletal muscle.

## Discussion

With‐no‐lysine 1 signaling plays an essential role for ion homeostasis through regulating the trafficking or activity of various membrane transport proteins 3, 5. While WNK1 is highly expressed in skeletal muscle 6, 7, the signaling pathways and physiological roles of WNK1 in skeletal muscle remain elusive. In this study, we demonstrate that insulin signaling phosphorylates WNK1 in a PI3K/Akt/TBC1D4‐dependent mechanism to promote cell surface abundance of GLUT4. This strongly implies that WNK1 is a novel mediator of insulin‐stimulated GLUT4 trafficking in skeletal muscle. Furthermore, WNK1 phosphorylation is significantly blunted in T2D *db/db* mice compared to that of wild‐type mice, indicating that WNK1‐regulated GLUT4 translocation may contribute to the impairment of glucose uptake in diabetic skeletal muscle.

With‐no‐lysine 1 is ubiquitously expressed at high levels in various tissues such as kidney, heart, and skeletal muscle 3, 6. While the mediators and mechanisms of ion transport in renal epithelial cells are relatively well characterized 3, 5, the biological roles of WNK1 in extrarenal tissue including skeletal muscle have yet been explored. Besides its role of ion homeostasis, WNK1 has been implicated in vesicle trafficking via regulating SNARE protein complex 13, 20, 21. Skeletal muscle accounts for approximately 80% of whole‐body glucose uptake after meals 8. GLUT4 is a major effector of insulin‐promoted glucose uptake in skeletal muscle. Insulin activates PI3K/Akt kinase to stimulate the translocation of GLUT4‐containing vesicles. WNK1 is a downstream substrate of Akt kinase 10, 11, 12, which phosphorylates WNK1 at threonine58 to regulate the cell surface expression of epithelial sodium channels and renal outer medullary potassium channels 10, 22. The present study shows that WNK1 regulates both basal and insulin‐stimulated translocation of GLUT4. The steady state of expression of GLUT4 at the plasma membrane is a balance between endocytosis and exocytosis from *de novo* synthesized proteins. Our results suggest that WNK1 regulates the basal membrane residence of GLUT4 via its constitutive translocation. WNK1 is a key regulator of both endocytosis and insertion of membrane proteins. Whether WNK1 regulates endocytosis of GLUT4 and, if so, the internalized GLUT4 is recycled back to plasma membrane should be examined in the future studies. On the other hand, insulin stimulates WNK1 to promote GLUT4 surface expressions via PI3K/Akt signaling cascades in cultured C2C12 myotubes and mouse skeletal muscle. TBC1D4, an AS, promotes GLUT4 translocation in response to insulin/Akt signaling. Eight phosphorylation sites of TBC1D4 have been identified 23, 24, and several kinases (Akt, serum and glucocorticoid‐regulated kinase 1, 5′ adenosine monophosphate‐activated protein kinase, and ribosomal S6 kinase 1) can phosphorylate TBC1D4 in distinct stimulus‐dependent manners 23. Both WNK1 and its N‐terminal fragment phosphorylates TBC1D4 *in vitro* kinase assay 13. However, the functional difference between the individual phosphorylation sites is yet ill‐defined. It is possible that WNK1 could directly and/or indirectly phosphorylate multiple sites of TBC1D4 including T588. Notably, it is previously presented that WNK1 forms a complex with TBC1D4 to stimulate GLUT1 trafficking in HEK293 cells 13. WNK1 regulates multiple ion channels and transporters via both kinase activity‐dependent and kinase activity‐independent mechanisms 2, 3, 5. In this study, insulin‐stimulated TBC1D4 phosphorylation was blunted by siRNA of WNK1 or kinase‐dead mutant (K233M) suggesting that the catalytic activity of WNK1 is critical for insulin‐promoted GLUT4 translocation. The functional relationship between individual TBC1D4 phosphorylation site and WNK1 regulation of GLUT4 awaits further future studies.

Translocation of GLUT4‐containing vesicles upon insulin stimulation is mediated via SNARE protein complex 8. Defects of SNARE protein complex and GLUT4 trafficking in skeletal muscle are strongly correlated with insulin resistance and diabetes 8, 13, 21. Phosphorylated Akt in skeletal muscle is decreased in type 2 diabetic *db/db* mice leading to insulin resistance in skeletal muscle. Our data show that WNK1 phosphorylation is significantly decreased in the context of total protein, but not at the levels of mRNA, in *db/db* mice when compared to wild‐type mice. Insulin stimulates Akt phosphorylation followed by WNK1 phosphorylation in a PI3K‐dependent mechanism 12, 25. In this respect, our data demonstrate the novel mechanism liaising WNK1 and insulin signaling effectors, Akt, TBC1D4, and GLUT4, with glucose transport of skeletal muscles in the development of T2D.

In summary, WNK1 is a highly expressed downstream substrate of InsR/PI3K/Akt pathway in mouse skeletal muscle. WNK1 promotes GLUT4 translocation via kinase‐dependent mechanism. In addition, compared with control mice, T2D *db/db* mice exhibit significant insulin resistance and decreased WNK1 phosphorylation. Altogether, our results provide a new perspective on WNK1 function beyond the regulation of ion homeostasis and offer a new molecular aspect of insulin resistance and the consequent pathogenesis of hyperglycemia in T2D.

## Author contributions

J‐HK, HK, and K‐HH designed the study, conducted the experiments, analyzed the data, and participated in writing the paper; JSC conducted the experiment for revision; KSP designed the study, contributed data analysis, and participated in writing paper; S‐KC and IDK designed and supervised the entire project and wrote the final manuscript. All authors read, commented, and approved the paper.

## Conflict of interest

The authors declare no conflict of interest.
